# Modulation of *N*-methyl-*N*-nitrosourea induced mammary tumors in Sprague–Dawley rats by combination of lysine, proline, arginine, ascorbic acid and green tea extract

**DOI:** 10.1186/bcr989

**Published:** 2005-01-31

**Authors:** M Waheed Roomi, Nusrath W Roomi, Vadim Ivanov, Tatiana Kalinovsky, Aleksandra Niedzwiecki, Matthias Rath

**Affiliations:** 1 Matthias Rath Research, Cancer Division, Santa Clara, California, USA

**Keywords:** antitumor effect, mammary tumors, *N*-methyl-*N*-nitrosourea, Sprague–Dawley rats

## Abstract

**Introduction:**

The limited ability of current treatments to control metastasis and the proposed antitumor properties of specific nutrients prompted us to examine the effect of a specific formulation (nutrient supplement [NS]) of lysine, proline, arginine, ascorbic acid, and green tea extract *in vivo *on the development of *N*-methyl-*N*-nitrosourea (MNU)-induced mammary tumors in rats.

**Methods:**

A single intraperitoneal dose of MNU was injected into each of 20 female Sprague–Dawley rats (aged 50 days) to induce tumors. Two weeks after MNU treatment, a time by which the animals had recovered from MNU-induced toxicity, the rats were divided into two groups. Rats in group 1 (*n *= 10) were fed Purina chow diet, whereas those in group 2 (*n *= 10) were fed the same diet supplemented with 0.5% NS. After a further 24 weeks, the rats were killed and tumors were excised and processed.

**Results:**

NS reduced the incidence of MNU-induced mammary tumors and the number of tumors by 68.4%, and the tumor burden by 60.5%. The inhibitory effect of NS was also reflected by decreased tumor weight; the tumor weights per rat and per group were decreased by 41% and 78%, respectively. In addition, 30% of the control rats developed ulcerated tumors, in contrast to 10% in the nutrient supplemented rats.

**Conclusion:**

These findings suggest that the specific formulation of lysine, proline, arginine, ascorbic acid, and green tea extract tested significantly reduces the incidence and growth of MNU-induced mammary tumors, and therefore has strong potential as a useful therapeutic regimen for inhibiting breast cancer development.

## Introduction

Breast cancer is the most prevalent cancer in women worldwide, excluding nonmelanoma skin cancer, and is the second leading cause of cancer deaths in women (following lung cancer) [1]. Once metastasis has occurred, the survival rate is drastically reduced to a median of 2–3 years; therapy is then aimed at controlling symptoms, prolonging survival and improving quality of life [2]. Unfortunately, the diagnostic criteria currently used to stage breast cancer often yield inaccurate findings with regard to metastasis. Analyses of bone marrow samples (not a routine procedure) have revealed the presence of disseminated cells in up to 40% of primary breast cancer patients without any clinical or histopathologic signs of metastasis. Circulating breast cancer cells in bone marrow are indicative of metastasis to such sites as bone, lung, and liver [3].

Cancer cells form tumors and spread by degrading the extracellular matrix (ECM) through various matrix metalloproteinases (MMPs). The activity of these enzymes correlates with the aggressiveness of tumor growth and the invasiveness of the cancer. Rath and Pauling [4] postulated that nutrients such as lysine and ascorbic acid could act as natural inhibitors of ECM proteolysis, and as such they have the potential to inhibit tumor growth and expansion. These nutrients may exert their antitumor effect through inhibiting MMPs and strengthening the connective tissue surrounding cancer cells (a 'tumor encapsulating' effect). Additionally, it has been suggested that, through inhibition of hyaluronidase, ascorbic acid can prevent metastases by preventing degradation of the ground substance surrounding the tumor.

In a previous study [5] we demonstrated the antiproliferative and anti-invasive potential of a specific formulation (nutrient supplement [NS]) of lysine, ascorbic acid, proline, and green tea extract on human breast cancer (MDA-MB 231), colon cell cancer (HCT 116), and melanoma (A2058) cell lines. NS also suppressed the growth of these tumors, without any adverse effects, in nude mice. In the present study we investigated the inhibitory effect of NS *in vivo *on development of *N*-methyl-*N*-nitrosourea (MNU)-induced mammary tumors in rats.

## Methods

### Animals

On arrival at our laboratory, 40-day-old pathogen free female Sprague–Dawley rats (Simonsen Laboratories, Gilroy, CA, USA) were housed in solid bottom cages with corncob bedding, at 22°C and 50% humidity, with a 12-hour light–dark cycle. The rats had free access to water and Purina rat chow diet. All animals were cared for in accordance with institutional guidelines for the care and use of experimental animals.

### Experiments

At day 50, all rats (*n *= 20) received a single dose of MNU 50 mg/kg intraperitoneally. (MNU, reagent grade, was obtained from Sigma, St. Louis, MO, USA). Two weeks after MNU treatment, a time by which the animals had recovered from MNU-induced toxicity, the rats were divided into two groups. Rats in group 1 (*n *= 10) were fed a pellet Purina chow diet (Purina Mills, Richmond, IN, USA), whereas those in group 2 (*n *= 10) were fed a pellet diet custom prepared by Purina containing the same diet but supplemented with 0.5% of the NS.

Body weight and diet consumption of the rats were monitored every week. Beginning 6 weeks after MNU administration, the rats were palpated every week for evidence of tumors. Dimensions (length × width) of the tumors were measured using a digital caliper, and the tumor burden was calculated using the following formula: 0.5 × length × width. Twenty-four weeks later the rats were killed by carbon dioxide asphyxiation and skinned; tumors were excised and a detailed necropsy was performed on each rat. Location, weight, and dimensions of excised mammary tumors were recorded. Tumors were processed for histologic examination, using criteria described by Russo and coworkers [6]. Briefly, histopathologic criteria used to determine malignancy were loss of tubular–alveolar pattern of the normal mammary gland; presence of large epithelial cells with increased nuclear–cytoplasmic ratio; stromal response by fibrosis and inflammatory cell infiltration; and necrosis and hemorrhage.

### Composition of the nutrient supplement

Stock solution of the NS (total weight 4.2 g) is composed of the following: 700 mg vitamin C (as ascorbic acid and as magnesium, calcium, and palmitate ascorbate), 1000 mg L-lysine, 750 mg L-proline, 500 mg L-arginine, 200 mg *N*-acetyl cysteine, 1000 mg standardized green tea extract, 30 mg selenium, 2 mg copper, and 1 mg manganese. Green tea extract, derived from green tea leaves, was obtained from US Pharma Lab (Newark, NJ, USA). The certificate of analysis indicates the following characteristics: total polyphenol 80%, catechins 60%, epigallocatechin-3-gallate (EGCG) 35%, and caffeine 1.0%.

### Statistical analysis

Results are expressed as means ± standard deviation for the groups. Data were analyzed by independent samples t-test.

## Results

### Tumor incidence and multiplicity

Of the 10 rats in the control group, nine developed at least one tumor; the total number of tumors in that group was 19. In contrast, five of the supplemented rats were completely free of tumors, and the total number of tumors in that group was six. As shown in Table 1, treatment with nutrients significantly reduced the incidence of MNU-induced mammary tumors and the number of tumors per rat (tumor multiplicity) by 68.4%.

### Tumor burden

Tumor burden (tumor length × width × 0.5) in MNU-induced mammary tumors was inhibited by nutrient synergy by 60.5% (*P *= 0.0001) as shown in Table 2. The mean tumor burden per rat for the control group was 18.3 cm^2^, in contrast to a mean tumor burden of 7.22 cm^2 ^in the nutrient supplemented group (*P *< 0.0001).

### Tumor weight

The inhibitory effect of NS was also reflected in a decreased tumor weight (Table 3). For example, tumor weight per rat and per group were decreased by 41% and 78%, respectively (*P *= 0.0001). The mean tumor weight per rat in the control group (4.34 g) was significantly greater (*P *= 0.002) than that in the supplemented group (0.97 g). The mean individual tumor weight also differed significantly between groups.

### Rat growth

We observed no significant difference (*P *= 0.62) in growth between groups over the period of study (Table 4).

### Tumor histology

**Figure 1:**
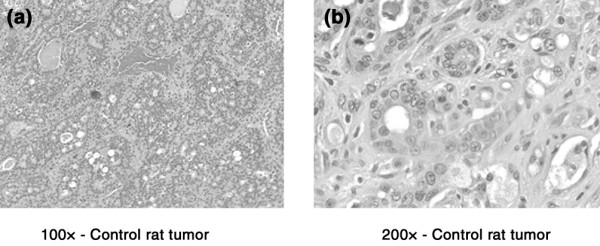
Histological slides showing examples of *N*-methyl-*N*-nitrosourea induced mammary tumors in Sprague–Dawley rats fed the control diet (hematoxylin and eosin). Original magnifications: **(a) **100× and **(b) **200×.

**Figure 2:**
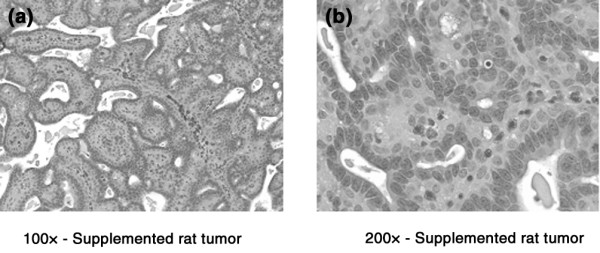
Histological slides showing examples of *N*-methyl-*N*-nitrosourea induced mammary tumors in Sprague–Dawley rats fed the supplemented diet (hematoxylin and eosin). Original magnifications: **(a) **100× and **(b) **200×.

The tumors that developed in the control group rats were all adenocarcinomas (Fig. 1). The lesions are rather cellular and consist of a proliferation of epithelial and stromal components; the epithelial elements range from reactive to malignant in nature. The majority of the tumor is adenomatous and exhibits features of florid sclerosing adenosis admixed with low-grade ductal carcinoma *in situ*. Focal areas exhibit features diagnostic of adenocarcinoma, including increased mitotic index, atypical mitotic figures, moderate to severe cytologic atypia, coagulative tumor cell necrosis, and jagged infiltrating margins. The lesion is well circumscribed in areas and is associated with a brisk host response composed of reactive stromal cells and a mixed inflammatory infiltrate. Angiogenesis is much more prominent than in the adenomas in the supplemented rats. In contrast, the tumors that developed in the nutrient supplemented rats were all adenomas (Fig. 2). The lesion is moderately cellular and consists of epithelial and stromal components. The overall cytoarchitectural features are indicative of a fibroepithelial lesion, such as a fibroadenoma. The epithelial cells exhibit mild to moderate cytologic atypia and a low mitotic index. The lesion is well circumscribed and exhibits prominent papillary architecture. The stromal and vascular proliferation is much less than that seen in the adenocarcinoma.

## Discussion

We chose to study the effect of a mixture of nutrients on MNU-induced mammary tumors in the Sprague–Dawley rat model because the histologic structure of mammary gland tumors in this animal closely resembles that of human mammary tumors. Induction of mammary carcinomas by MNU in female rats is one of the most frequently used animal models for the investigation of breast carcinogenesis and mammary tumor treatment [7-9]. In contrast to mouse lesions, which are primarily alveolar, rat mammary tumors are predominantly ductal, as are human ones [9], and the most highly malignant rat tumors share some features with human intraductal and infiltrating ductal carcinomas [8]. It has been reported that the MNU model has several advantages, such as reliability of tumor induction, organ site specificity, tumor of ductal origin and predominantly carcinomatous histopathologic characterization, and the ability to examine tumor initiation and promotion processes [10]. Generally MNU-induced mammary carcinomas are aggressive and locally invasive.

The results of the present study demonstrate significant inhibition of mammary tumor incidence and multiplicity in Sprague–Dawley female rats by supplementation with 0.5% of the nutrient mixture (which contains ascorbic acid, lysine, proline, and epigallocatechin gallate). Furthermore, rats that consumed the nutrient supplemented diet exhibited decreased growth of MNU-induced mammary tumors and tumor burden compared with rats fed control diets. The numerous tumors in the control rats not only were larger but also had characteristics diagnostic of adenocarcinoma, including increased mitotic index and prominent angiogenesis, in contrast to the few, small adenomas with low mitotic index found in the nutrient treated rats.

Although the mechanism underlying the reduced tumor size in tumor bearing rats was not identified in this experiment, these findings are consistent with our previous *in vitro *studies that demonstrated significant inhibition of angiogenic and invasive parameters in human breast cancer cell lines MDA MB-231 and MCF-7. Expression of vascular endothelial growth factor, MMP secretion, and matrix invasion by these breast cancer cells were dramatically inhibited in a dose-dependent manner by the combined effect of the nutrients in this mixture [11]. Matrix invasion can be controlled by inhibition of MMP expression, as well as by increasing connective tissue strength and stability, contributing to the 'encapsulation' of the tumor. Optimization of synthesis and structure of collagen fibrils depends upon hydroxylation of proline and lysine residues in collagen fibers. It is well known that ascorbic acid is essential for the hydroxylation of these amino acids and that it regulates collagen synthesis at the transcriptional level.

Inhibitory and chemopreventive effects in malignant cell lines of some of the individual nutrients composing the NS have been reported in both clinical and experimental studies. Ascorbic acid has been shown to have growth inhibitory and antineoplastic activities in human mammary tumor bearing mice [12]. In addition, low levels of ascorbic acid have been reported in cancer patients [13-15]. Green tea extract is another potent anticancer agent that has been reported to have a growth inhibitory effect against certain human cancer cell lines, especially breast cancer [16-18]. For example, both *in vitro *and animal studies of the effect of green tea extract on breast cancer revealed suppressed xenograft size and tumor vessel density and suppression of cell proliferation [19].

Furthermore, studies conducted before the clinical onset of breast cancer found that increased green tea consumption was associated with improved prognosis in stage I and II breast cancers, as well as decreased numbers of axillary lymph node metastases in premenopausal women, suggesting significant chemopreventative potential [20]. Utilizing the Sprague–Dawley rat model, researchers showed that EGCG reduced tumor burden and number of invasive tumors, and drastically increased the mean latency to the initial tumor in mammary tumor bearing rats [21].

Our previous *in vitro *studies demonstrated that the anticancer effect of a mixture of ascorbic acid, proline, lysine, and EGCG on several cancer cell lines in tissue culture studies was greater than that of the individual nutrients [5]. Furthermore, in contrast to chemotherapy, which causes indiscriminate cellular and ECM damage, previous studies showed that cell morphology was not affected even at the highest concentrations of this nutrient mixture, demonstrating that this formulation is safe to cells.

## Conclusion

The results of the present study showed that the specific nutrient mixture of lysine, proline, arginine, ascorbic acid, and green tea extract tested significantly inhibited the incidence, as well as the growth, of MNU-induced mammary tumors. Although clinical trials are necessary to assess the antitumor ability of the tested nutrient mixture on cancer patients, the results of this study suggest strong potential for its use as a therapeutic regimen for inhibiting breast cancer development.

## Abbreviations

ECM = extracellular matrix; EGCG = epigallocatechin-3-gallate; MMP = matrix metalloproteinase; MNU = *N*-methyl-*N*-nitrosourea; NS = nutrient supplement.

## Competing interests

This research was funded by Matthias Rath Inc.

## Authors' contributions

MWR carried out tumor burden, tumor weight, and tumor multiplicity laboratory studies. NR assisted in laboratory studies. VI designed the study. TK drafted the manuscript and performed the statistical analysis. AN and MR conceived the study and participated in its coordination. All authors read and approved the final manuscript.

## Figures and Tables

**Table 1:** Tumor incidence and multiplicity for control and supplemented rats

Rat group	0 Tumors	1 Tumor	2 Tumors	3 Tumors	4 Tumors	5 Tumors	Total number of Tumors
Control group (*n *= 10)	1	4	3	0	1	1	19
Supplemented group (*n *= 10)	5	4	1	0	0	0	6

**Table 2:** Mean tumor burden per rat and tumor burden per group

Rat group	Total tumor burden per group	Mean tumor burden per rat
Control group (*n *= 10; 19 tumors)	183.2 cm^2^	18.3 ± 1.3 cm^2^
Supplemented group (*n *= 10; six tumors)	72.2 cm^2^	7.22 ± 1.8 cm^2^

The mean tumor burden per rat is expressed as mean ± standard deviation. The differences between groups were significant (*P *< 0.0001).

**Table 3:** Mean tumor weight per rat and per group

Rat group	Total tumor weight per group	Mean tumor weight per rat	Mean individual tumor weight
Control group (*n *= 10; 19 tumors)	43.38 g	4.34 ± 1.5 g	2.3 ± 0.8 g
Supplemented group (*n *= 10; six tumors)	9.63 g	0.97 ± 2.6 g	0.73 ± 0.5 g
Significance	*P *= 0.002	*P *= 0.002	

**Table 4:** Mean growth of control and supplemented rats

Rat group	Mean initial weight	Mean end weight
Control group (*n *= 10)	143 ± 4 g	422 ± 24 g
Supplemented group (*n *= 10)	148 ± 5 g	415 ± 37 g
